# ﻿Three new species of the genus *Rhogadopsis* Brèthes (Hymenoptera, Braconidae, Opiinae) from South Korea

**DOI:** 10.3897/zookeys.1214.132694

**Published:** 2024-10-10

**Authors:** Yunjong Han, Cornelis van Achterberg, Hyojoong Kim

**Affiliations:** 1 Animal Systematics Laboratory, Department of Biological Science, Kunsan National University, Gunsan, 54150, Republic of Korea Kunsan National University Gunsan Republic of Korea; 2 Naturalis Biodiversity Center, P.O. 9517, 2300 RA Leiden, Netherlands Naturalis Biodiversity Center Leiden Netherlands

**Keywords:** Identification, key, new combination, new species, new synonym, parasitoid wasp, South Korea

## Abstract

Three new species of *Rhogadopsis* Brèthes, 1913 (*R.clausulata***sp. nov.**, *R.obliqoides***sp. nov.** and *R.setosipunctata***sp. nov.**) are described and illustrated. *Rhogadopsisunicarinata* (Fischer, 1959) is a new combination and a new synonym of *R.mediocarinata* (Fischer, 1963), **syn. nov.** An identification key to the species of *Rhogadopsis* known from South Korea is provided.

## ﻿Introduction

The cosmopolitan subfamily Opiinae Blanchard, 1845, comprises 39 genera and approximately 2100 described valid species, consisting generally of small (1–5 mm body length) parasitoid wasps (Yu et al. 2016). It contains koinobiont parasitoid wasps that mainly parasitize leaf-mining and fruit-infesting cyclorrhaphous dipterous larvae. The classification of Opiinae genera is still under discussion and fluctuating, primarily due to uncertainties regarding some genera like *Opius* Wesmael, 1835, and *Eurytenes* Foerster, 1863 (Wharton 1987, 1988, 1997; Li et al. 2013; Wharton and Norrbom 2013). Papp (1989) reported Opius (Rhogadopsis) parvungula (Thomson) from North Korea (a junior synonym of *R.reconditor* (Wesmael, 1835)). Han and Kim (2023) reported *R.obliqua* Li & van Achterberg from South Korea. However, the Korean specimen of *R.obliqua* differs from the holotype by having the second and third metasomal tergites distinctly sculptured medially, the vein CU1b of the fore wing comparatively short, and vein the m-cu of the hind wing absent. In addition, *Opiusmediocarinatus* (Fischer, 1963), reported from the Korean Peninsula, has been recombined into the genus *Rhogadopsis* (Chen et al. 2016) and should be included in this study.

We treat the genus *Rhogadopsis* Brèthes, 1913, as a valid genus separate from *Opius* Wesmael, 1835, as was proposed by Li et al. (2013). Three new species are described and illustrated, and an identification key to the Korean *Rhogadopsis* is provided below.

## ﻿Material and methods

Specimens of *Rhogadopsisclausulata* sp. nov., *R.setosipunctata* sp. nov. and the holotype of *R.obliqoides* sp. nov. were collected in a Malaise trap, while the paratype of *R.obliqoides* sp. nov. was collected by using a net to sweep the herbal vegetation. For identification of the subfamily Opiinae, see van Achterberg (1990, 1993, 1997); for references to Opiinae, see Yu et al. (2016).

Morphological terminology follows van Achterberg (1988, 1993), including the abbreviations for the wing venation. Measurements were taken as indicated by van Achterberg (1988): the maximum length and width of a body part were taken unless otherwise indicated. The length of the mesosoma was measured from the anterior border of the mesoscutum to the apex of the propodeum and of the first tergite from the posterior border of the adductor to the medio-posterior margin of the tergite.

Observations, photographic images, and descriptions were made either with a Leica DMC2900 digital camera or with a Leica M205 C microscope (Leica Geosystems AG) or with a digital camera on a Zeiss Stereo Discovery V12 with AxioVision SE64 Rel. 49.1 software for stacking. The photos from the Leica system were stacked with Helicon Focus v. 7 software (Helicon Soft, Kharkiv, Ukraine). After stacking, illustrations were created using Adobe Photoshop CS5.1.

The holotype and paratype of *Rhogadopsisclausulata* sp. nov. are deposited in the Naturalis Biodiversity Center (**RMNH**) at Leiden, and the type specimens of *R.obliqoides* sp. nov. and *R.setosipunctata* sp. nov. are deposited in the Kunsan National University (**KSNU**) at Gunsan.

## ﻿Systematics

### ﻿Genus Rhogadopsis

Taxon classificationAnimaliaHymenopteraBraconidae

Brèthes, 1913

465581A5-FF8B-5A9C-AE23-2EF7704BC7F6

Rhogadopsis Brèthes, 1913: 44; Shenefelt 1975: 1212; Wharton 1987: 66 (as subgenus Lissosema). Type species (by monotypy): Rhogadopsisminiacea Brèthes, 1913.
Lissosema Fischer, 1972: 359. Type species (by original designation): Opiusparvungula Thomson, 1895 (= Opiusreconditor Wesmael, 1835; Broad et al. 2016: 291).

#### Diagnosis.

Propodeum with a distinct (but often short) medio-longitudinal carina anteriorly (Figs 5, 11, 34); hypoclypeal depression variable; mandible symmetrical or nearly so (Figs 7, 8, 19, 32); dorsope absent; vein m-cu of fore wing usually gradually merging into vein 2-CU1, and linear with vein 2-M or nearly so (Fig. 14); if angled then hind wing comparatively wide, vein 1r-m of hind wing less oblique and 0.6–1.0× as long as vein 1-M (Figs 2, 15, 27); medio-posterior depression of mesoscutum variable (Figs 4, 17, 29); anterior groove of metapleuron usually crenulate (Figs 12, 16, 28); precoxal sulcus largely present, crenulate (Figs 12, 16, 28); vein CU1b of fore wing completely present (Figs 2, 14, 26).

#### Distribution.

Palaearctic, Oriental, Nearctic and Afrotropical regions.

#### Biology.

Parasitoids of mining dipterous larvae of the family Agromyzidae (*Agromyza* Fallen, 1810, *Amauromyza* Hendel, 1931, *Calycomyza* Hendel, 1931, *Cerodontha* Rondani, 1861, *Liriomyza* Mik, 1894, *Metopomyza* Enderlein, 1936, *Napomyza* Westwood, 1840, *Phytomyza* Fallen, 1810).

### ﻿Key to Korean species of the genus *Rhogadopsis* Brèthes

**Notes.** The number of included species for Korea is based on the National Institute of Biological Resources (2019), Chen et al. (2016) and this study.

**Table:** 

1	Medio-posterior depression of mesoscutum present (Figs 17, 29); antenna of ♀ with 23–26 segments and of ♂ with 26 segments; first metasomal tergite more widened posteriorly and 0.7–0.9× as long as its apical width (Figs 18, 30); second tergite variable	**2**
–	Medio-posterior depression of mesoscutum absent (Fig. 4); antenna of both sexes with 28–41 segments; first metasomal tergite less widened posteriorly and 1.0–1.6× longer than its apical width; second tergite smooth	**3**
2	Mesoscutum and scutellum finely punctate (Fig. 29); antero-dorsal area of mesopleuron brown; second metasomal suture and second metasomal tergite smooth (Fig. 31); vein m-cu of fore wing interstitial or nearly so; vein m-cu of hind wing present (Fig. 27)	***R.setosipunctata* Han & van Achterberg, sp. nov.**
–	Mesoscutum and scutellum smooth; mesopleuron entirely black; second metasomal suture distinct crenulate dorsally; second tergite at least basally distinctly sculptured (Fig. 18); vein m-cu of fore wing distinctly postfurcal; vein m-cu of hind wing absent (Fig. 15)	***R.obliqoides* Han & van Achterberg, sp. nov.**
3	Hypoclypeal depression absent (Figs 7, 8); first tergite about as long as wide apically (Fig. 5); vein m-cu of fore wing angled with vein 2-CU1 (Fig. 2)	***R.clausulata* Han & van Achterberg, sp. nov.**
–	Hypoclypeal depression present; first tergite 1.2–1.6× as long as wide apically; vein m-cu of fore wing gradually merging in vein 2-CU1	**4**
4	Clypeus nearly parallel-sided, slightly narrowed laterally, approximately 4.0× wider than high and largely smooth; first tergite 1.2–1.3× longer than wide apically; face finely punctate	***R.reconditor* (Wesmael, 1835)**
–	Clypeus trapezoid or narrow semi-circular, distinctly narrowed laterally, 2.5–3.0× wider than high and punctate; first tergite 1.3–1.6× longer than its apical width; face rather coarsely punctate; [antenna of both sexes with 32–41 segments]	***R.unicarinata* (Fischer, 1959), comb. nov. [= *R.mediocarinata* (Fischer, 1963), syn. nov.**]

### ﻿ Rhogadopsis clausulata

Taxon classificationAnimaliaHymenopteraBraconidae

Han & van Achterberg sp. nov.

6584862C-2139-500E-9989-2B7EFBAEB74B

https://zoobank.org/9C9127E9-34AA-4F65-865A-776AAB870CB9

Figs 1 , 2–10 , 11–12

#### Type material.

***Holotype***, • ♀ (RMNH), “South Korea: Kangwondo, Cuncheon, Nam-myeon, Hudong-li, MT [= Malaise trap], 17.viii.–5.ix.2003, 37°44'N 127°35'E, P. Tripotin, RMNH’12”. ***Paratype***: • 1 ♀ (RMNH), “South Korea: Kangwondo, Cuncheon, Magogli, along Hongchen river, 70 m, 12.vi.–11.vii.2004, 37°44'N 127°35'E, P. Tripotin, RMNH’12”.

#### Diagnosis.

Antennal scape and pedicel yellowish-brown, but flagellum dark brown (Figs 9, 10); hypoclypeal depression absent (Figs 7, 8); mandible with a rather basal lamella below at base (Fig. 8; invisible in anterior view); notauli largely absent on disc (Fig. 4); mesoscutum and scutellum shiny, smooth and largely glabrous; medio-posterior depression of mesoscutum absent (Fig. 4); pterostigma wide triangular and narrowed apically (Fig. 2); vein m-cu of fore wing distinctly postfurcal; second and following tergites dark brown, except light pattern posteriorly (Fig. 5); setose part of ovipositor sheath shorter than first metasomal tergite and hardly protruding beyond apex of metasoma (Fig. 6).

#### Description.

Holotype, female; length of body 3.9 mm, of fore wing 3.6 mm.

***Head*.** Antenna with 33 segments and 1.1× longer than body; third antennal segment 3.0× longer than its width (Figs 9, 10); eye 1.3× longer than temple in dorsal view; vertex smooth and frons glabrous, punctate, setose; median keel on face smooth and shiny; clypeus 2.0× wider than its maximum height, moderately setose, rather flat in lateral view and its ventral margin slightly protruding medio-apically; hypoclypeal depression absent (Fig. 7); maxillary palp 1.1× longer than height of head; malar sulcus present (Figs 7, 8); occipital carina absent dorsally; mandible symmetrical with a rather basal lamella below at base in lateral view (Fig. 8).

**Figure 1.**
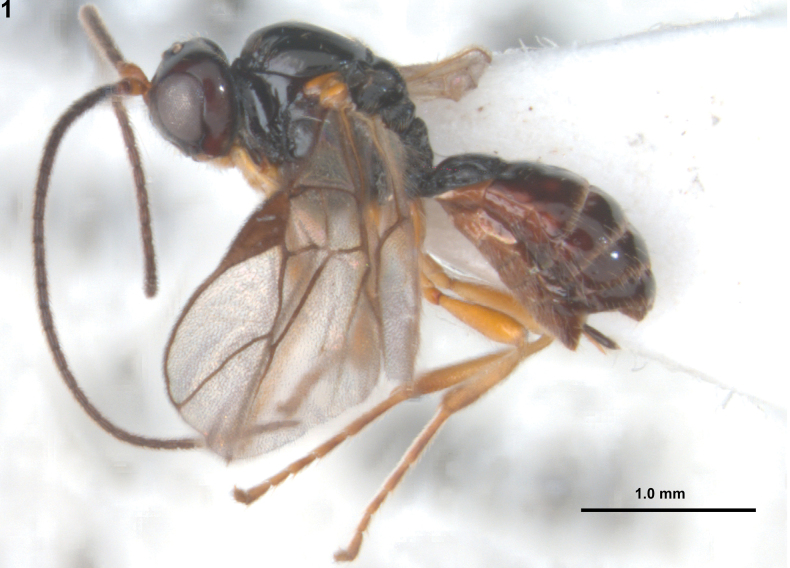
*Rhogadopsisclausulata* Han & van Achterberg, sp. nov., holotype, ♀, South Korea, habitus, lateral.

**Figures 2–10.**
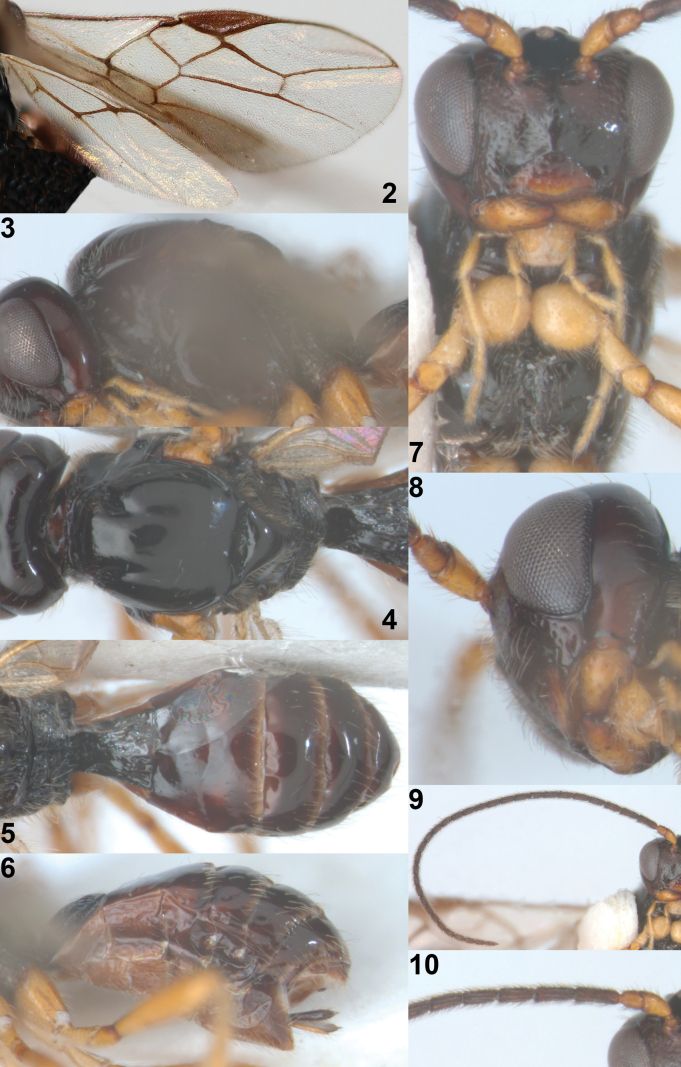
*Rhogadopsisclausulata* Han & van Achterberg, sp. nov., holotype, ♀, South Korea **2** wings **3** mesosoma, lateral **4** mesosoma, dorsal **5** propodeum and metasoma, dorsal **6** metasoma, lateral **7** head anterior and mesonotum, antero-ventral **8** mandible, latero-ventral **9** antenna **10** base of antenna.

***Mesosoma*.** Mesosoma in lateral view 1.3× longer than its height; pronope large and elliptical (Fig. 4); propleuron shiny and smooth; pronotal side largely shiny and smooth, but postero-ventral area sculptured; mesopleuron smooth, including narrow precoxal sulcus (Fig. 12); epicnemial area crenulate ventrally, remaining area smooth; mesopleural sulcus crenulate; anterior groove of metapleuron crenulate; metapleuron shiny, reticulate-rugose and moderately setose; notauli absent on disc but with pair of short deep impressions anteriorly (Fig. 4); mesoscutum and scutellum shiny, smooth and sparsely setose; medio-posterior depression of mesoscutum absent (Fig. 4); scutellar sulcus widened medially and distinctly crenulate; propodeum coarsely reticulate-rugose with a short medio-longitudinal carina and diverging oblique two transverse carinae (Figs 4, 5).

***Wings*.** Fore wing (Fig. 2): pterostigma wide, triangular and gradually narrowed apically; vein 1-M almost straight; vein 1-SR+M straight; vein 3-SR linear with vein r, 1.5× longer than vein 2-SR; vein 2-SR oblique; vein SR1 nearly straight; r:3-SR:SR1 = 3:29:57; vein m-cu distinctly postfurcal and 4× longer than vein 2-SR+M; vein cu-a postfurcal; vein CU1b short (Fig. 2). Hind wing: vein m-cu only pigmented basally; vein 1r-m 0.6× as long as vein 1-M; vein 2-M pigmented.

***Legs*.** Length of hind femur 3.4× its maximum width (Fig. 1).

***Metasoma*.** First tergite as long as its apical width, its surface shiny, reticulate-rugose and convex medially in lateral view (Figs 5, 6); dorsope absent, but dorsal carinae strongly developed and separated posteriorly (Figs 4, 5); following tergites smooth and moderately setose posteriorly (Fig. 5); second metasomal suture obsolescent dorsally; setose part of ovipositor sheath 0.3× as long as first tergite and 0.05× as long as fore wing, and slightly protruding beyond apex of metasoma (Fig. 6).

#### Colour.

Generally black or blackish-brown (Fig. 1); scape and pedicel of antenna and ventral half of clypeus yellowish-brown; mandible, tegulae and legs light brown; palpi pale yellowish; posterior bands of third to seventh tergites yellowish; pterostigma and veins of wings brown; wings hyaline.

#### Variation.

The paratype is very similar to the holotype, but antenna with 36 segments, length of body 4.1 mm, and of fore wing 3.6 mm, medio-longitudinal carina of propodeum distinct and half as long as propodeum.

#### Distribution.

South Korea.

#### Biology.

Unknown.

#### Etymology.

Named after the closed hypoclypeal depression: “*clausus*” is Latin for shut or closed.

**Figures 11, 12.**
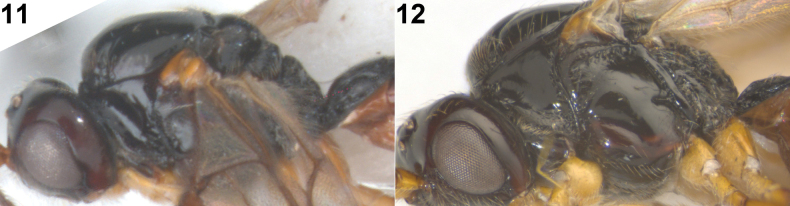
*Rhogadopsisclausulata* Han & van Achterberg, sp. nov., ♀, South Korea **11** holotype mesosoma, lateral **12** paratype mesosoma, lateral.

#### Remarks.

This new species runs to *Rhogadopsis* Brèthes by having a distinct medio-longitudinal carina on the propodeum, a complete vein CU1b of fore wing, a symmetrical mandible and a wide hind wing with less oblique vein 1r-m of hind wing, 0.6× as long as vein 1-M (van Achterberg 2023). It is similar to *Rhogadopsisunicarinata* (Fischer) because of the lack of the medio-posterior depression of the mesoscutum, the short vein r of the fore wing and the distinct medio-longitudinal carina of the propodeum (Chen et al. 2016). It differs from the latter by the robust first tergite (slender in *R.unicarinata*), lack of the hypoclypeal depression (wide), head mainly black (more or less yellow), absence of precoxal sulcus (present medially), vein m-cu of fore wing straight (curved) and the very large pronope (absent).

### ﻿ Rhogadopsis obliqoides

Taxon classificationAnimaliaHymenopteraBraconidae

Han & van Achterberg sp. nov.

E5CB45D2-D4F8-53A7-8594-C5928B1FD4BD

https://zoobank.org/9B8F875A-A596-4A8B-B946-6E7C0B21F713

Figs 13 , 14–24

#### Type material.

***Holotype***, • ♀ (KSNU), “South Korea: Baekgye-ro, Okryong, Gwangyang, Jeonnam, 10.ix.–24.ix.2019, 35°01'41"N 127°36'51"E, MT [= Malaise trap], Hyojoong Kim leg., KSNU”. ***Paratype***, • 1 ♂ (KSNU), “South Korea: Yeoseo-ri, Cheongsan-myeon, Wando, Jeonnam, 2.vii.2020, 33°59'15.1"N 126°55'04.6"E, SW [=collected by sweeping], Hyojoong Kim leg., KSNU”.

**Figure 13.**
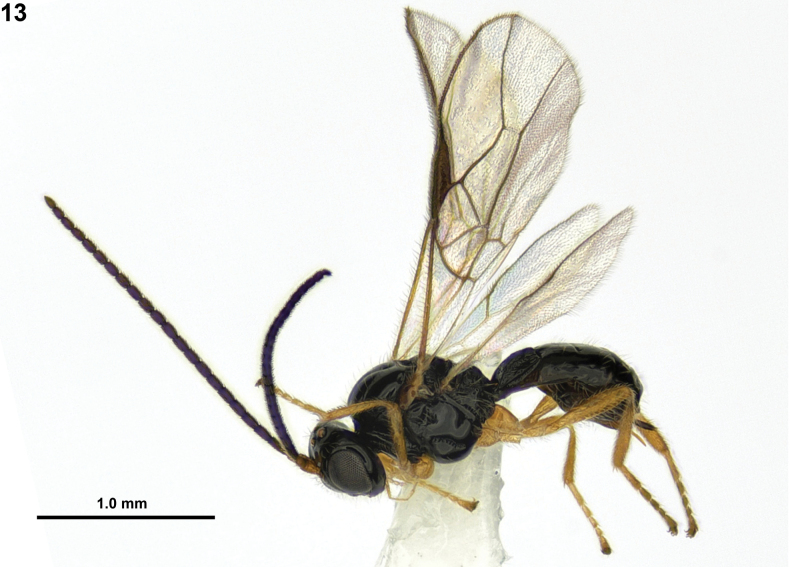
*Rhogadopsisobliqoides* Han & van Achterberg, sp. nov., holotype, ♀, South Korea, habitus, lateral.

#### Diagnosis.

**Figures 14–24.**
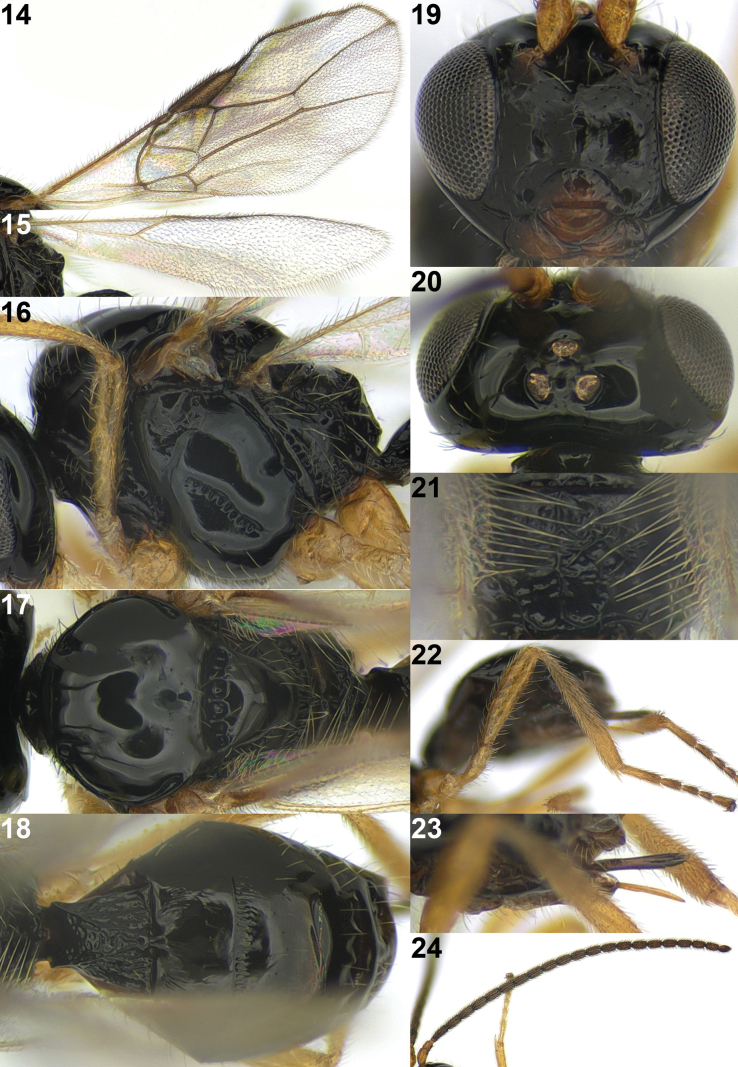
*Rhogadopsisobliqoides* Han & van Achterberg, sp. nov., holotype, ♀, South Korea **14** fore wing **15** hind wing **16** mesosoma, lateral **17** mesosoma, dorsal **18** metasoma, dorsal **19** head, anterior **20** head, dorsal **21** propodeum **22** hind leg **23** ovipositor and sheath **24** antenna.

Second and third metasomal tergites largely smooth except for some faint sculpture anteriorly of both tergites (Fig. 18); hypoclypeal depression distinct (Fig. 19); precoxal sulcus crenulate (Fig. 16); medio-posterior depression of mesoscutum present (Fig. 17); first tergite reticulate-rugose without transverse carinae (Fig. 18); vein m-cu of fore wing linear with veins 2-CU1 and 2-M (Fig. 14); vein 1r-m of hind wing 0.7× as long as vein 1-M; vein m-cu of hind wing absent.

#### Description.

Holotype, female; length of body 2.1 mm, of fore wing 2.2 mm.

***Head*.** Antenna with 23 segments and as long as body; third segment 2.8× longer than its width, 1.1× longer than fourth segment (Fig. 24); eye in dorsal view 3.2× longer than temple (Fig. 20); vertex and frons shiny, smooth and sparsely setose; clypeus 2.1× wider than its maximum height (Fig. 19); clypeus rather convex with setae and its ventral margin almost straight; hypoclypeal depression distinct (Fig. 19); maxillary palp 0.8× longer than height of head; malar sulcus present; occipital carina interrupted dorsally; mandible triangular and gradually widened basally (Fig. 19).

***Mesosoma*.** Mesosoma in lateral view 1.3× longer than its height; pronope deep, large and round (Figs 17, 20); mesopleuron smooth but precoxal sulcus oblique and crenulate (Fig. 16); epicnemial area smooth dorsally; mesopleural sulcus smooth; anterior groove of metapleuron crenulate, ventral area faintly rugose and remainder area smooth; notauli absent on disc except for both deep and short impressions anteriorly (Fig. 17); mesoscutum shiny, smooth and sparsely setose along imaginary notaulic courses and around medio-posterior depression; scutellum shiny and smooth; medio-posterior depression of mesoscutum round (Fig. 17); scutellar sulcus widened and distinctly crenulate; propodeum coarsely reticulate-rugose with a medio-longitudinal carina and two oblique transverse carinae (Fig. 21).

***Wings*.** Fore wing (Fig. 14): pterostigma triangular, gradually narrowed apically; veins 1-M and SR1 curved; veins 1-SR+M and 2-SR straight; vein 3-SR 1.7× longer than vein 2-SR; r:3-SR:SR1 = 5:36:57; vein m-cu distinctly postfurcal and twice as long as vein 2-SR+M; vein CU1b rather long (Fig. 14); first discal cell closed. Hind wing (Fig. 15): vein m-cu absent; vein 1r-m 0.7× as long as vein 1-M.

***Legs*.** Length of hind femur 4.1× its maximum width (Fig. 22); hind femur with rather long and tibia with medium-sized setae.

***Metasoma*.** First tergite 0.9× as long as its apical width, its surface shiny, irregularly and densely rugose and convex medially in lateral view (Fig. 18); dorsope absent, but dorsal carinae strongly developed (Fig. 18); second metasomal suture distinct; second tergite largely smooth but anteriorly striate-rugose; third tergite largely smooth but densely and shortly striate-rugose anteriorly; following tergites smooth and with few setae posteriorly; setose part of ovipositor sheath 0.7× as long as first tergite and 0.1× as long as fore wing, slightly protruding beyond apex of metasoma (Fig. 23).

#### Colour.

Generally black (Fig. 13); scape and pedicel of antenna brown; antenna, ventral half of clypeus and mandible dark brown; palpi pale yellowish; legs and tegular yellowish-brown; veins and pterostigma greyish; wings subhyaline.

#### Distribution.

South Korea.

#### Biology.

Unknown.

#### Etymology.

The name is a combination of the specific name “*obliqua*” and “*oides*” (Latin for resembling) because the new species is similar to *R.obliqua* Li & van Achterberg.

#### Remarks.

This species runs to *Rhogadopsisobliqua* Li & van Achterberg in the key of Li et al. (2013), because of having the antenna of ♀ with only about 23 segments, clypeus 2.1× wider than its maximum height, the medio-posterior depression of mesoscutum distinctly impressed, the propodeum with a short medio-longitudinal carina, the wide hind wing with less oblique vein 1r-m of hind wing, 0.7× as long as vein 1-M, the third metasomal tergite largely smooth, ventro-posterior crenulate groove of pronotal side, the medio-posterior depression of mesoscutum distinctly developed and the precoxal sulcus crenulated. However, it differs by having the second metasomal tergite more or less sculptured (smooth in *R.obliqua*), the lack of vein m-cu of hind wing (presented as unpigmented trace), vein 1-M of fore wing distinctly curved (slightly curved), vein m-cu of fore wing twice as long as vein 2-SR+M (1.5 times longer) and hind tibia of female wider and conspicuously setose (narrower and sparse setose).

### ﻿ Rhogadopsis setosipunctata

Taxon classificationAnimaliaHymenopteraBraconidae

Han & van Achterberg sp. nov.

DCB9E89F-6983-5069-BFAF-CA7229611F6F

https://zoobank.org/A5B24C03-7A2D-4F9D-B580-CBC6B6842796

Figs 25 , 26–37

#### Type material.

***Holotype***, • ♀ (KSNU), “South Korea: Gonggeun-ri, Gonggeun, Hoengseong, Gangwon, 19.vii.–6.viii.2019, 37°33'58.3"N 127°57'54.9"E, MT [= Malaise trap], Hyojoong Kim leg., KSNU”.

#### Diagnosis.

Mesoscutum and scutellum finely punctate and setose (Fig. 29); antenna of ♀ with 26 segments; hypoclypeal depression distinct (Fig. 32); precoxal sulcus crenulate (Fig. 28); antero-dorsal area of mesopleuron brown; medio-posterior depression of mesoscutum present (Fig. 29); vein m-cu of fore wing interstitial (Fig. 26) or nearly so; first metasomal tergite 0.7× as long as its apical width (Fig. 30) and smooth as following metasomal tergite.

**Figure 25.**
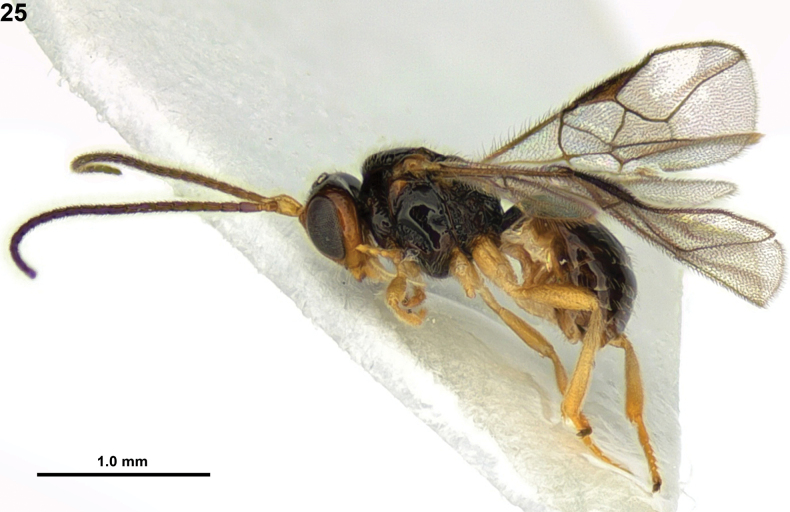
*Rhogadopsissetosipunctata* Han & van Achterberg, sp. nov., holotype, ♀, South Korea, habitus, lateral.

#### Description.

Holotype, female; length of body 2.4 mm, of fore wing 2.4 mm.

***Head*.** Antenna with 26 segments and as long as body (Fig. 37); third segment 2.7× longer than its width, 1.1× longer than fourth segment; eye in dorsal view 2.2× longer than temple (Fig. 33); vertex and face shiny, punctate and densely setose; clypeus 2.3× wider than its maximum height (Fig. 32); clypeus in lateral view rather convex with long setae and its ventral margin slightly concave; hypoclypeal depression distinct (Fig. 32); maxillary palp 0.7× as long as height of head; occipital carina interrupted dorsally; mandible triangular and gradually widened basally (Fig. 32).

***Mesosoma*.** Mesosoma in lateral view 1.3× longer than its height; pronope deep, large and round; mesopleuron smooth but precoxal sulcus oblique, robust and crenulate (Fig. 28); epicnemial area crenulate ventrally; mesopleural sulcus smooth; anterior groove of metapleuron crenulate; metapleuron area coarsely rugose ventrally with setae and remaining area smooth; notauli absent on disc except deep and short impressions anteriorly (Fig. 29); mesoscutum and scutellum shiny, punctate and densely setose; medio-posterior depression of mesoscutum elliptical (Fig. 29); scutellar sulcus robust and distinctly crenulate; propodeum reticulate-rugose, with a short medio-longitudinal carina anteriorly and two oblique transverse carinae, remaining area shiny and smooth (Figs 29, 34).

**Figures 26–37.**
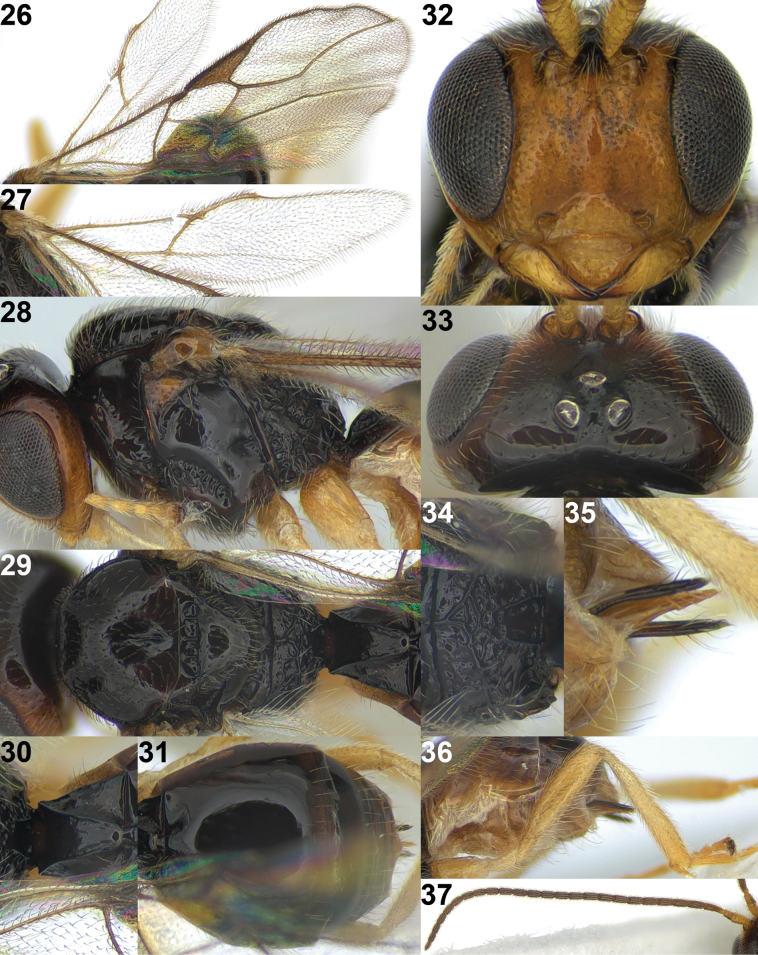
*Rhogadopsissetosipunctata* Han & van Achterberg, sp. nov., holotype, ♀, South Korea **26** fore wing **27** hind wing **28** mesosoma, lateral **29** mesosoma, dorsal **30** first metasomal tergite, dorsal **31** metasoma, dorsal **32** head, anterior **33** head, dorsal **34** propodeum **35** ovipositor and sheath **36** hind leg **37** antenna.

***Wings*.** Fore wing (Fig. 26): pterostigma triangular, gradually narrowed apically; veins 1-M and SR1 curved; vein 1-SR 0.3× as long as vein 1-M; vein 1-SR+M sinuate; vein r 0.7× as long as vein 1-SR; vein 3-SR 1.3× longer than vein 2-SR; r:3-SR:SR1 = 7:32:54; veins m-cu, cu-a interstitial; first subdiscal cell closed. Hind wing (Fig. 27): wide; vein m-cu faintly pigmented; vein 1r-m 0.7× as long as vein 1-M.

***Legs*.** Length of hind femur 3.6× its maximum width (Fig. 36).

***Metasoma*.** First tergite 0.7× as long as its apical width, its surface shiny, smooth and convex medially in lateral view; dorsope absent, dorsal carinae strongly developed and reaching apex of tergite (Figs 29, 30); second metasomal suture absent; second tergite shiny, smooth, with a pair of oblique depressions anteriorly; following tergites shiny, smooth with posterior row of setae; setose part of ovipositor sheath 0.7× as long as first tergite and 0.09× as long as fore wing, slightly protruding beyond apex of metasoma (Fig. 35).

#### Colour.

Generally black to dark brown (Fig. 25); scape of antenna, mandible and legs yellowish-brown; antenna, face, tegulae and mesopleuron antero-dorsally brown; palpi pale yellowish; veins and pterostigma brown to dark brown; wing membrane subhyaline.

#### Distribution.

South Korea.

#### Biology.

Unknown.

#### Etymology.

Named after the uniformly punctate and setose face, mesoscutum and scutellum; “*punctus*” is Latin for “point”, and “*setosus*” is Latin for “with setae”.

#### Remarks.

This new species fits well in the genus *Rhogadopsis* because of the short medio-longitudinal carina on the propodeum anteriorly, the symmetrical mandible, the complete vein CU1b of fore wing, the wide hind wing with less oblique vein 1r-m of hind wing 0.7× as long as vein 1-M and anterior groove of metapleuron crenulated. The species is unique among the East Palaearctic and Northeast Oriental species because of the punctate mesoscutum and scutellum, the smooth first tergite with coarse dorsal carinae up to the apex of the tergite and the lack of vein 2-SR+M of the fore wing (a result of the subinterstitial vein m-cu).

## Supplementary Material

XML Treatment for Rhogadopsis

XML Treatment for Rhogadopsis clausulata

XML Treatment for Rhogadopsis obliqoides

XML Treatment for Rhogadopsis setosipunctata

## ﻿Additional information

### Author ORCIDs

Yunjong Han https://orcid.org/0000-0003-2757-7785

Cornelis van Achterberg https://orcid.org/0000-0002-6495-4853

Hyojoong Kim https://orcid.org/0000-0002-1706-2991

### Data availability

All of the data that support the findings of this study are available in the main text.
